# Functional connectivity changes resemble patterns of pTDP-43 pathology in amyotrophic lateral sclerosis

**DOI:** 10.1038/srep38391

**Published:** 2016-12-08

**Authors:** Ines Schulthess, Martin Gorges, Hans-Peter Müller, Dorothée Lulé, Kelly Del Tredici, Albert C. Ludolph, Jan Kassubek

**Affiliations:** 1 Department of Neurology, University of Ulm, Ulm, Germany; 2 Clinical Neuroanatomy, Department of Neurology, University of Ulm, Germany

## Abstract

‘Resting-state’ fMRI allows investigation of alterations in functional brain organization that are associated with an underlying pathological process. We determine whether abnormal connectivity in amyotrophic lateral sclerosis (ALS) in *a priori*-defined intrinsic functional connectivity networks, according to a neuropathological staging scheme and its DTI-based tract correlates, permits recognition of a sequential involvement of functional networks. ‘Resting-state’ fMRI data from 135 ALS patients and 56 matched healthy controls were investigated for the motor network (corresponding to neuropathological stage 1), brainstem (stage 2), ventral attention (stage 3), default mode/hippocampal network (stage 4), and primary visual network (as the control network) in a cross-sectional analysis and longitudinally in a subgroup of 27 patients after 6 months. Group comparison from cross-sectional and longitudinal data revealed significantly increased functional connectivity (*p* < 0.05, corrected) in all four investigated networks (but not in the control network), presenting as a network expansion that was correlated with physical disability. Increased connectivity of functional networks, as investigated in a hypothesis-driven approach, is characterized by network expansions and resembled the pattern of pTDP-43 pathology in ALS. However, our data did not allow for the recognition of a sequential involvement of functional connectivity networks at the individual level.

It is a major challenge of current neuroscience to understand functional brain organization on the basis of its structural and functional connectivity, together with the corresponding pathological substrate in the diseased brain^1^. Here, we address this aim in amyotrophic lateral sclerosis (ALS), a rapidly progressive motor neuron disease^2^. Structural connectivity measures of fiber bundles in a hypothesis-guided approach to address ALS-related pathology have provided evidence of considerable tract damage in a sequential progression^3^. These results supported *in vivo* the proposed neuropathological model of a corticofugal axonal spreading pattern of phosphorylated transactive response DNA-binding protein 43 kDa (pTDP-43) pathology beginning in the agranular motor cortex and then progressing to precerebellar nuclei towards prefrontal and postcentral neocortices as well as striatal neurons and finally reaching the temporal lobe, including the hippocampus^4^,^5^. Fractional anisotropy (FA) as a common metric of diffusion tensor imaging (DTI) has consistently revealed ALS-associated patterns of white matter damage in the corticospinal tract (CST) as the DTI correlate of ALS stage 1^3^,^6^,^7^,^8^, and DTI changes have been suggested as a potential surrogate marker for ALS^9^.

Previous studies on the functional connectivity in ALS reported abnormalities^10^,^11^,^12^ and offer new insights into the underlying pathological processes. According to the model of progression of TDP-43 pathology^4^,^5^,^13^, we introduced a hypothesis-guided approach and investigated *a priori*-defined functional connectivity networks as follows: motor network corresponding to neuropathological stage 1, brainstem network corresponding to stage 2, ventral attention network corresponding to stage 3, the default mode/hippocampal network, which includes the parahippocampal cortex and hippocampal formation as major nodes^14^, corresponding to stage 4. These functional connectivity networks are well-defined^15^,^16^ and were chosen according to the pathological^4^ and the DTI-based *in-vivo* model^3^,^17^. We additionally included the primary visual network as a control network in which, according to the pathoanatomical model^5^ and the DTI-based *in vivo* staging model^3^,^17^, we expected no functional connectivity alterations^16^. The functional networks cover functionally connected brain regions that are prone to develop ALS-associated pathology in a sequential fashion. We hypothesized that the functional network-associated regions become sequentially involved which may result in network-based abnormal functional connectivity patterns.

Using ‘resting-state’ (rs-)fMRI, we compared the functional network organization in these four intrinsic connectivity networks between ALS patients and healthy controls over time to determine whether possible functional connectivity alterations permit recognition of abnormal BOLD synchronization. In addition, we used tractwise fractional anisotropy statistics^18^, a DTI-based fiber tracking technique to determine tract-averaged FA-values, for correlation analysis between structural and functional connectivity measures.

## Results

### Patterns of increased functional connectivity in ALS patients

**Figure 1:**
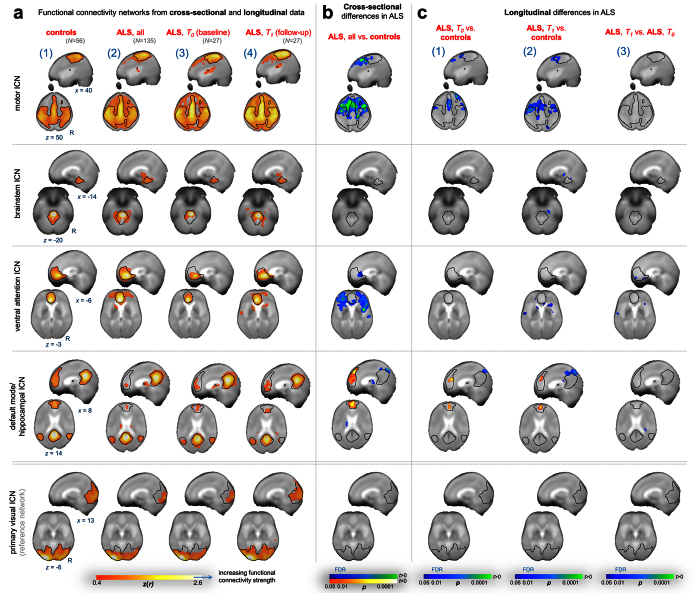
Functional connectivity alterations in ALS. (**a**) BOLD synchronization illustrated as sagittal and axial connectivity heat maps showing voxel-wise Fisher’s *r*-to-*z* transformed correlation coefficients (thresholded for |*z(r*)| ≥ 0.4) for which the fMRI BOLD signal was correlated with the seed-voxel in M1 (motor network, **upper row**), the midbrain (brainstem network, **second row**), ventral striatum (ventral attention network, **third row**), the posterior cingulate cortex (default mode network/hippocampal, **fourth row**), and V1 in the occipital pole (primary visual network used as reference network, **bottom row**) for controls (**1**), ALS patients cross-sectionally (**2**), and ALS patients measured over time (**3**). *z(r*)-values indicate the strength of correlation as an indirect measure of functional connectivity. (**b**) Cross-sectional analysis indicated increased functional connectivity in ALS patients (*N* = 71) compared with controls (*N* = 27). However, the default mode/hippocampal network (**lfourth row**) revealed, besides increased (**cool colors**) functional connectivity, areas of regional decrease (**hot colors**) of functional connectivity in the prefrontal cortex. (**c**) Longitudinal information indicated statistically significant increases of functional connectivity in ALS patients (*N* = 14) at initial (**1**) and follow-up MRI acquisition (**2**) as compared with controls, consistent with the cross-sectional data (**b**). Analysis in ALS patients over time revealed statistically significant clusters (voxel-wise paired *t*-test) indicating increased functional connectivity between initial (ALS, *T*_*0*_) and follow-up (ALS, *T*_*1*_) MRI measurement (**3**). The patterns of increasing functional connectivity presented as network expansions as illustrated by the delineations (**black solid lines**, **a2**–**4**, **B**,**C**) corresponding to the functional connectivity maps (i.e., networks) for controls as shown in (**b**[**1**]). (**b**,**c1–3**) Clusters indicating statistically significant group effects (*p* < 0.05, voxel-wise *t*-tests) were corrected at a 5% false discovery rate (FDR)-level with further cluster-wise correction discarding small cluster (<343 mm^3^). (**a**,**b**,**c**) All results are shown in MNI stereotaxic space (cubic 1 mm grid) overlaid on the averaged study-specific echo planar imaging (EPI) template. Slice positions in each row (**a1**) are identical for panels associated with the motor (**upper row**), brainstem (**second row**), ventral attention (**third row**), default mode/hippocampal network (**fourth row**), and primary visual network (reference network, **bottom row**), respectively.

The functional connectivity patterns associated with the motor, brainstem, ventral attention, and default mode/hippocampal intrinsic connectivity network revealed increased connectivity maps in ALS patients (*N* = 135) compared with controls (*N* = 56) (Fig. 1a,b). These findings indicated a network expansion over time, as demonstrated for the MRI scans in a subgroup of patients (*N* = 27) compared with the 6-month follow-up scans in these patients (Fig. 1c). The patterns of abnormal BOLD synchrony at the group level were observed in the aforementioned four networks but were not observed, in accordance with the hypothesis, in the primary visual network which was used as control network (Fig. 1, **bottom row**). Patterns of significantly increased functional connectivity (Fig. 1b) were in agreement with previous studies^11^. Notably, the significant clusters were predominantly localized ‘outside’ the controls’ functional connectivity maps (see delineation of the controls’ network in Fig. 1), thereby, indicating network expansions^19^.

Thus, according to our pathoanatomy-associated hypotheses, motor, brainstem, ventral attention and default mode/hippocampal intrinsic connectivity networks appear to be involved in the ALS-associated pathoanatomical process. This pattern of increasing functional connectivity was in general agreement with the longitudinal data (Fig. 1c) for group-wise statistical comparisons between ALS patients at baseline (*N* = 27) vs. controls (*N* = 56) and ALS patients at follow-up (*N* = 27) vs. controls (*N* = 56). The networks were shown to expand over time (i.e. about 6 months on average); however, direct pairwise group comparison between ALS patients at baseline vs. follow-up showed only a tendency towards network expansion over time since most of the clusters did not survive our strict thresholding (Fig. 1c). However, our ‘resting-state’ fMRI data did not permit definition of a model of a sequential ‘dysfunctional’ spread since we could not provide, with sufficient specificity, the identification of abnormal network-based functional connectivity alterations at the individual level.

Remarkably, the group differences in the default mode/hippocampal network between ALS patients (*N* = 135) vs. controls, on the other hand, indicated significantly decreased functional connectivity of the medial prefrontal cortex which is a major node within the default mode/hippocampal network (Fig. 1b). The motor network appeared to expand towards anterior and posterior regions (Fig. 1), which resembled the distribution patterns of TDP-43 pathology^4^. In order to rule out the potential effects of the MRI protocols, we randomly split our large cohort of ALS patients (*N* = 135) into a test data set (*N* = 68) and a validation data set (*N* = 67), each compared with controls (*N* = 56). We could validate our findings by demonstrating very similar effects for both datasets, in full agreement with the reported results.

### Correlations between CST impairment and functional connectivity

**Figure 2:**
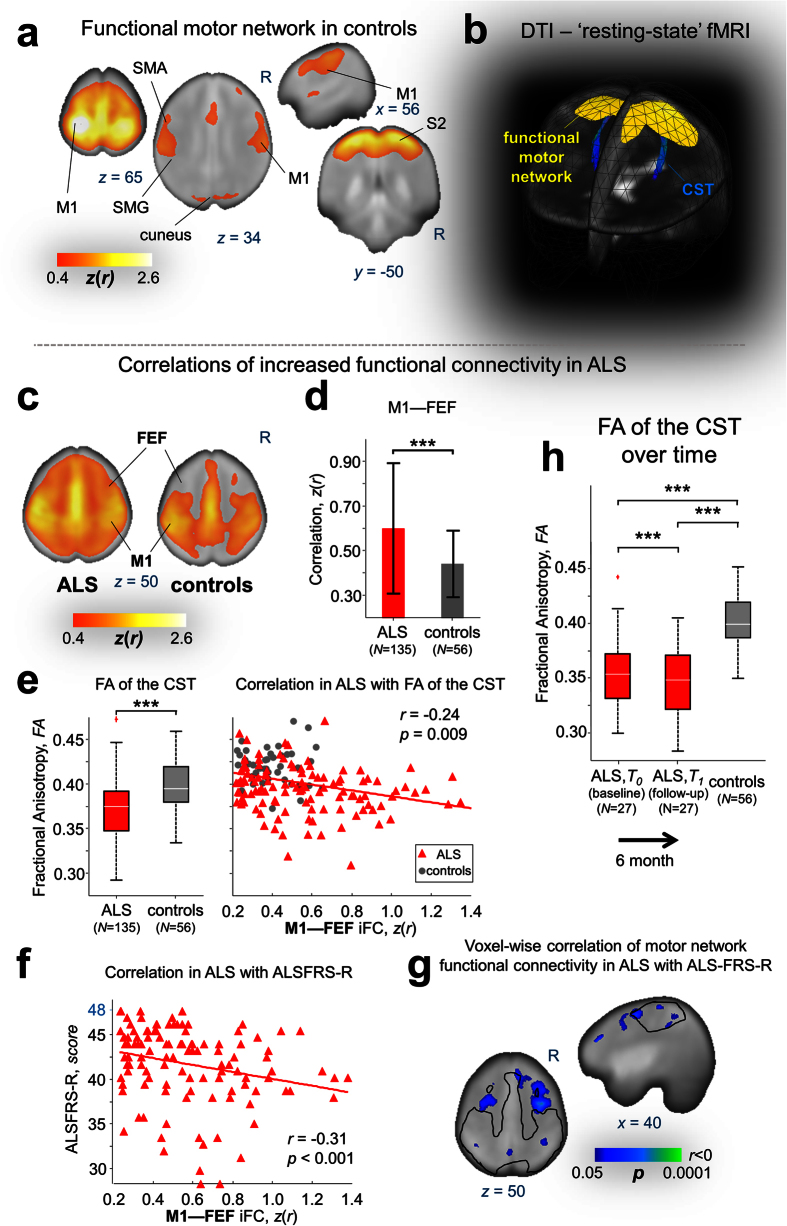
Correlations of motor network functional connectivity in ALS. (**a**) Orthogonal slices showing heat maps of the motor intrinsic functional connectivity network for the control group (*N* = 56, for details see Figure 1a) and (**b**) its corresponding 3-D representation (**yellow**) together with the cortico spinal tract (CST, **blue**) on the averaged fractional anisotropy (FA)/(*b* = 0)-template on a cubic 1 mm grid in MNI stereotaxic space. (**c**) Most representative axial slice (*z* = 50) showing functional motor network in ALS patients (*N* = 135) and controls (*N* = 56), indicating network expansion in ALS predominantly towards frontal areas. (**d**) Primary motor cortex (M1) correlations (measured by BOLD synchronization) in ALS patients and controls as exemplified for region-to-region functional connectivity between primary motor cortex (M1) and frontal eye field (FEF), i.e. M1—FEF functional connectivity (*t*-test; *t* = 4.53, *p* < 0.0001). (**e**) Significantly reduced FA values in ALS patients compared with controls indicating microstructural impairment of the CST (Mann-Whitney-*U* test; *z* = 6.37; ***p < 0.0001) and corresponding negative correlation (Spearman rank order *r* = 0.24; *p* = 0.009; corrected) with M1—FEF functional connectivity in ALS. (**f**) Spearman rank order correlation (r = −0.31; p = 0.0006) of M1—FEF functional connectivity with revised ALS Functional Rating Scale (ALSFRS-R). (**g**) Cool color map showing significantly Spearman rank order voxel-wise correlations (*p* < 0.05, cluster-wise corrected) of M1 connectivity with ALSFRS-R. This pattern largely overlaps with the functional network expansion in ALS (cf. Fig. 1b, **upper row**), indicating that functional connectivity increases with increasing physical impairment. Delineations correspond to the motor network of the controls. (**h**) Damage in the CST in ALS patients worsens over time as indicated by significantly reduced FA values in ALS patients (*N* = 27) compared with controls (*N* = 56) at initial (Mann-Whitney-*U* test; *z* = 3.63; *p = 0.0003) and follow-up MRI scans (Mann-Whitney-*U* test; *z* = 4.09; **p < 0.0001) and in the comparison over time (Wilcoxon signed rank test; *z* = 3.19; ***p = 0.0017). (**a**, **c**, **g**) Results are overlaid on the study-specific EPI template (1 mm cubic grid in MNI space). Abbreviations: SMA, Somatosensory motor area; SMG, supra marginal gyrus; S2, Secondary somatosensory cortex; iFC, intrinsic functional connectivity.

The results of DTI-based analyses are summarized in Table 1. In accordance with previously published studies^3^,^11^,^20^, tract-based spatial statistical mapping of FA for the investigated fiber tracts, i.e. the CST (Fig. 2b), corticopontine and corticorubral tract, corticostriatal pathway, and the proximal portion of the perforant pathway, demonstrated significantly decreased FA-values in all ALS patients (*N* = 135) compared with healthy controls (*N* = 56) with an exception of the reference pathway in area 5 of the corpus callosum. It is of note that the significance level revealed a marked gradient with increasing *p*-values beginning from the CST (*p* < 10^−6^, corresponding to stage 1^3^), corticopontine and –rubral tract (*p* < 10^−5^, stage 2^3^), striatal pathway (*p* < 10^−4^, stage 3^3^) to the proximal portion of the perforant pathway (*p* < 10^−2^, stage 4^3^) which is in a general line of agreement with the proposed DTI-based *in vivo* staging scheme, as previously published^17^,^21^. Pair-wise comparison in ALS patients over time (*N* = 27) indicated considerably progressive white matter damage in the CST (Fig. 2h) and corticopontine tract/corticorubral tract after approximately 6 months, whereas the striatal pathway and the proximal portion of the perforant pathway did not reach statistical significance (Table 1). The reference tract did not show any significant FA-value alterations in both cross-sectional and longitudinal data as hypothesized (Table 1).

Cross-sectional and longitudinal data of ALS patients revealed significant correlations between the FA values and functional connectivity measures within the motor (Fig. 2a–e), brainstem, ventral attention, and default mode/hippocampal network. Notably, correlation analysis exhibited positive correlation, i.e., FA decreases with decreasing functional connectivity. The corresponding clusters were predominantly located ‘inside’ the controls’ networks in regions known as major nodes of the respective networks. These findings indicated that the burden of structural impairment was associated with a beginning loss of connectivity between major nodes of the respective network.

### Correlations between physical impairment and functional connectivity

Physical impairment in ALS patients was measured using the ALS-FRS-R score and subjected to the correlation analysis with both functional connectivity measures and structural impairment (FA values) of the CST. We demonstrated clusters indicating significantly negative correlations between the ALS-FRS-R score and functional connectivity in the motor (Fig. 2f,g), default mode/hippocampal, and brainstem network in ALS patients (*p* < 0.05, FDR corrected with further cluster wise correction). The clusters (shown in cool colors in Fig. 1b, **upper row**) indicated functional network expansions in ALS patients; these patterns of increased functional connectivity consistently resembled the pattern of significant voxel-wise correlation between motor network functional connectivity and physical disability (Fig. 2g).

Taken together, the pattern of increasing functional connectivity maps was significantly correlated with increasing physical impairment, and major nodes of the respective networks tended to decrease functional interaction with each other in the course of the disease. In addition, FA of the CST was significantly correlated with the ALS-FRS score in ALS patients (*N* = 135, Spearman’s rank order *r* = 0.44, *p* < 0.0001).

## Discussion

Using a hypothesis-guided MRI approach to investigate the network-based functional connectivity in ALS *in vivo*, this study demonstrated an increasing pattern of functional connectivity network expansions over time and signs of functional decoupling between major nodes of all investigated networks that were correlated with both DTI-based impairment in the CST and with increasing physical disability. It appears that hyper-connectivity is an imaging correlate in ALS that presents with a pattern of functional network expansions following the distribution of TDP-43 pathology. In addition, increasing functional networks may accompany the development of a disconnection syndrome presenting as functional discoupling within major nodes of the respective networks^22^. However, abnormal patterns of synchronous BOLD activity did not allow the recognition of a sequential ‘dysfunctional’ spreading at the individual level due to methodological limitations.

Almost all neurons that develop TDP-43 pathology are projection neurons with long axons and include a broad spectrum of motor and non-motor cell types^5^. Therefore, hyper-connectivity seems to be more than a correlate of a widespread degeneration of GABA-ergic neurons causing a loss of the inhibitory influence^11^. If functional alterations (‘hyper-connectivities’) are caused by a loss of inhibitory influences, then this calls for future multimodal studies on ‘resting-state’ fMRI including a complementary neuroimaging modality such as positron emission tomography^23^. It is of note that increased functional connectivity is not ALS-specific and has been demonstrated in patients with Parkinson’s disease^24^ as a possible early manifestation prior to the development of a functional disconnection syndrome that parallels cognitive decline later in the course of the disease^19^.

Structural damage occurs early during the course of ALS^7^ and is linked to abnormal brain functioning in a ‘more-with-less’ fashion as measured by functional connectivity^25^. The *in vivo* transfer of the proposed neuropathological staging scheme^3^ was recently addressed by a graph theory-based computational model of white matter impairment in ALS^21^. The authors demonstrated a strongly interconnected component of the brain network likely serving as an anatomical infrastructure facilitating pTDP-43 spread, as the computational evidence of disease spread in ALS to be directed and constrained by the topology of the anatomical brain network, in support of the results of the present study.

Increased (‘hyper-’)connectivity might be interpreted as stronger synchronous BOLD fluctuations^26^ and have been discussed in ALS as disease-specific pathological processes that might result from a loss of the inhibitory influence^11^. One correlate of abnormal BOLD activity in both cross-sectional and longitudinal analyses of our study might be pathologically increased firing patterns in a ‘denial-of-service’ fashion, i.e., additionally recruited brain areas excessively communicating with each other are very limited or ultimately no longer capable of functional interaction within their designated networks^19^. This finding is consistent with the notion of impaired dynamic up- and down-regulation of functional network activity^10^ that might be crucial for normal brain functioning^27^. A second straightforward explanation of increased functional connectivity for the networks might be an early compensatory or adaptive response to disease-related pathology^22^. Additional resources may be allocated in recruiting further brain structures by reorganizing functional networks in order to bypass affected regions until the neuronal reserve is exhausted when a critical cell loss is reached^26^.

The group comparison between ALS patients and controls provided additional evidence of decreased functional coupling between major nodes of the networks, as supported by both the significant correlations with CST impairment, i.e., the higher the burden of CST impairment the lower the functional coupling and by the decreased functional connectivity in the default mode/hippocampal network in agreement with^12^. A decoupling of memory-related brain regions as part of the default mode/hippocampal networks is believed to be linked to cognitive deficits, as suggested in many previous studies^26^ and also specifically manifested in ALS patients in clinical assessments^28^. Functional abnormalities within the brainstem network may support the neuropathological evidence of the involvement of the inferior olivary complex, which might be seen in a principle agreement with oculomotor abnormalities such as a saccadized smooth pursuit known to be a behavioral correlate of damaged pre-cerebellar circuits^29^.

The study is not without limitations. Post-mortem validation of the pTDP-43 pathology was not available in the studied patients. Hence, our results show increased connectivity in networks that match regions involved pathologically, but the analyses do not allow conclusions about sequential ordering of network dysfunction. Moreover, functional connectivity as analysed in this study does not specify the direction of information flow in the underlying network connectivity, and the contribution of inhibitory and excitatory neuronal coupling to functional connectivity measures cannot be disentangled^30^. We investigated intra-network but not inter-network functional connectivity in networks defined by a seed-voxel. However, seed-based descriptions only measure the connectivity with respect to the reference voxel (‘seed’) and, hence, may fail to characterize the full functional connectome. This methodological issue might be overcome by graph-based network analysis^21^, building upon region-to-region connectivity analysis based on a fine-grain voxel-wise parcellation of the cortex^31^ as the subject of future studies. In ALS patients, patterns of increased functional connectivity were consistently observed in the hypothesis-guided analysis of the four networks, i.e. motor, brainstem, ventral attention and default mode/hippocampal network in the cross-sectional data and received support from the longitudinal data. However, pair-wise statistical comparison between ALS patients over time, i.e. baseline vs. follow-up “resting-state” fMRI assessment, revealed only few small clusters after strict correction for multiple comparisons. This is most probably due to methodological issues, i.e. (1) the relatively low number (*N* = 27) of follow-up MRI in ALS patients, (2) the relatively short time interval of about 6 months between measurements using ‘resting-state’ MRI. On the other hand, our statement is markedly strengthened by the demonstrated correlations with both (1) tract-based axonal fiber loss (i.e. FA of CST) and (2) measures of physical impairment (i.e. ALSFRS-R). The by default limited spatial resolution of “resting-state” data and the limited signal-to-noise ratio of the BOLD signal required the common preprocessing step of spatial smoothing in order to increase the signal-to-noise ratio at the expense of blurring some white matter structure into the grey matter. Hence, the clusters cannot be fully disentangled between grey and white matter. Moreover, the human brain’s complex cortical folding differs dramatically between individuals^32^. This high inter-subject variability of the cortical convolution cannot be overcome by normalization into the common stereotaxic MNI space. Subjects’ head motion-induced artefacts might contribute to the rs-fMRI signal and are believed to produce spurious correlation patterns^33^ which should be considered in any interpretation of our results. However, we think this potential contributing factor can be ruled out because (1) in-scanner head motion during rs-fMRI data acquisition were corrected using standardized procedures^34^ and (2) our longitudinal data supported increased functional connectivities attributed to the ongoing pathological process. In this study, data from two MRI protocols were merged into the statistical analysis. Possible differences between scanning protocols do not appear to considerably contribute to the results at the group level; in order to minimize potential influences resulting from different protocols, we used the same ratio of patients to controls for both protocols so that there was no obvious need for calculating correction metrics for merging the scanning protocols. The relatively small number of 27 follow-up measurements is a further limitation of the present study; however, longitudinal studies in ALS are rare and a pose a challenge owing to rapid disease progression and the physical load during the MRI scans^35^. Our ‘resting-state’ fMRI data did not permit definition of a model of a sequential ‘dysfunctional’ spread since we could not provide, with sufficient specificity, the identification of abnormal network-based functional connectivity alterations at the individual level. The lack of classification of abnormal functional connectivity at the individual level is most probably due to the nature of BOLD fluctuations that are an indirect measure for functional connectivity and are technically constrained by the limited signal-to-noise ratio and limited spatial resolution which both could not be fully overcome by the standardized preprocessing pipeline.

In conclusion, the functional connectivity substrate of the underlying ALS-related pathology appears to be characterized by an extension of particular functional networks (as investigated in the hypothesis-driven approach), i.e. increased functional connectivity relative to controls that possibly parallels gradually decreasing connectivity between major nodes of functional networks which is correlated with the burden of axonal damage in the CST and increasing physical disability. These network expansions remarkably resembled the pattern of TDP-43 pathology in ALS. However, our ‘resting-state’ fMRI data from the four specifically investigated networks did not allow for the recognition of a sequential spreading at the individual level. Together with the proposed *in vivo* model of a DTI-based tract-wise progression pattern in ALS, an enhanced understanding of the associated network-based functional alterations may contribute to the development of new models of ALS-specific functional brain architecture. If these findings could supply the groundwork for potential new disease-modifying strategies has to await future studies.

## Methods

### Subjects and clinical characterization

All patients included in the study provided written informed consent for the MRI protocol according to institutional guidelines. The study was approved by the Ethics Committee of the University of Ulm, Germany (reference #19/12) and was performed in accordance with the ethical standards laid down in the 1964 Declaration of Helsinki and its later amendments.

Patients with ALS (*N* = 135) underwent initial (baseline) MRI scans at enrollment (for details of the scanning protocols, see *MRI acquisition* below), together with standardized clinical-neurological and routine laboratory examinations. The diagnosis of all patients was made by a motor neuron disease specialist according to the recently revised El Escorial diagnostic criteria. None of the ALS patients had any history of other neurological or psychiatric disorders. A daily dose of 100 mg riluzole was administered to 132 out of 135 patients (98%). Follow-up investigations were conducted in a subgroup of ALS patients (*N* = 27) after approximately 6 months (median 192 days, range 132–367).

MRI data and demographic characteristics of matched healthy controls (*N* = 56) for comparison were obtained from a normal database. None had a history of neurological or psychiatric disease or other medical conditions. Detailed features of ALS patients and controls are summarized in Table 2.

### MRI acquisition

The study utilized two different protocols for whole-brain based rs-fMRI and DTI data acquisition. The data for 67 ALS patients (out of 135) with follow-up measurement for 14 patients and 28 controls were acquired at a 3 T MR scanner (Allegra Siemens Medical, Erlangen, Germany). Functional rs-fMRI data were obtained using a BOLD contrast sensitive gradient echo echo-planar sequence (30 transversal slices, gap 1 mm, 3 × 3 × 4 mm^3^ voxels, 64 × 64 × 30 matrix, TE 30 ms, TR 2000 ms, flip angle 90°, 120 volumes). The DTI study protocol consisted of 49 gradient directions, including one *b*0 gradient direction (no gap, 2.2 mm^3^ iso-voxels, 96 × 128 × 52 matrix, TE 85 ms, TR7600 ms, *b*1000 s/mm^2^).

The data for the remaining 68 ALS patients (out of 135) with follow-up measurement for 13 patients and 28 controls were acquired at a 1.5 Tesla clinical MRI scanner (Symphony, Siemens Medical, Erlangen, Germany). Functional rs-fMRI data were obtained using a BOLD contrast sensitive gradient echo echo-planar sequence (30 transversal slices, 3.27 × 3.27 × 3.00 mm^3^ voxels, 64 × 64 × 30 matrix, TE 28 ms, TR 3080 ms, flip angle 90°, 120 volumes). The DTI study protocol consisted of 52 gradient directions, including four *b*0 gradient directions (no gap, voxelsize 2.0 × 2.0 × 2.8 mm3, 128 × 128 × 64 matrix, TE 95 ms, TR 8000 ms, b1000 s/mm^2^).

### MRI data analysis

Functional and structural imaging data were analysed using the *Tensor Imaging and Fiber Tracking* (TIFT) software package^36^. Warping for rs-fMRI and DTI images to Montreal Neurological Institute (MNI) stereotaxic standard space was applied by use of a study-specific template according to^18^,^34^.

### rs-fMRI data processing and statistical analysis

Preprocessing of rs-fMRI data followed conventional practice^33^,^34^,^37^. Briefly, this included (1) resampling to an isotropic 1 mm grid, (2) head-motion correction, (3) MNI normalization, (4) spatial smoothing, (5) demeaning and detrending, (6) temporal bandpass filtering, and (7) discarding first 10 volumes.

To insure sufficient image quality, all echo-planar imaging (EPI) volumes were visually inspected for proper registration. None of the subjects had to be excluded prior to analysis due to artefacts.

The data were resampled resulting in identical voxel resolution by means of a nonparametric k-nearest neighbor interpolation in order to combine both MRI protocols^38^ in a common isotropic 1 mm grid of a 256 × 256 × 256 matrix for further processing steps^34^. All volumes were motion-corrected within runs using a rigid body transformation in all directions (six degrees of freedom) with respect to the first volume in order to correct for physical motion confounding factors. An iterative semi-manual landmark-based deformation approach applied to each subject individually^34^ was used to normalize all EPI volumes into the MNI stereotaxic standard space by additionally using a study-specific EPI template computed from all subjects included in the analysis (*N* = 191). This MNI deformation procedures additionally incorporate possible grey-matter atrophy as a confounding factor on functional connectivity analysis^19^. Spatial filtering was applied to the EPI series by using a 8 mm 3-dimensional full-width at half maximum (FWHM) Gaussian blur filter in order to increase signal-to-noise ratio at the expense of blurring some small-sized axonal fiber bundles into gray matter structures. The chosen kernel size of 8 mm is a common choice^34^ and equals about twice the in-plane resolution for both MRI protocols as an appropriate choice according to the ‘matched filter’ design. Prior to spatial smoothing, we used a CSF mask^39^ obtained from the study-specific EPI template in order to attenuate the confounding CSF signal and to prevent blurring of the CSF signal into adjacent gray matter structures. The functional image time series were demeaned and detrended to correct for possible scanner-drifts and were then bandpass filtered using a 6^th^-order Butterworth bandpass filter design with cut-off frequencies in the range of 0.01 < f < 0.08 Hz. Finally, the first 10 EPI volumes were discarded due to the transient filter response in order to correct for possible scanner oscillations at the beginning of the functional MRI protocol as well as to allow the subjects to adapt to the experimental condition (e.g. noisy environment). No regressions of nuisance waveforms were performed since any set of regressors (such as WM and CSF signal) may remove highly structured signal^37^.

The networks were chosen in a hypothesis-guided approach, i.e. motor network corresponding to neuropathological stage 1, brainstem network corresponding to stage 2, ventral attention network corresponding to stage 3, the default mode/hippocampal network corresponding to stage 4, and primary visual network used as control network.

These five large-scale correlation maps were computed using the seed-based approach, i.e., (1) motor (voxel-seed: motor cortex; MNI coordinates (x y z): -25-37 65), (2) brainstem (midbrain; 2-31-20), (3) ventral attention (ventral striatum; -11 13 0), (4) default mode/hippocampal network (posterior cingulate cortex; 0-55 26), and (5) primary visual network (V1; -28-93-7). Finally, the resulting brain maps (*r*-values) were voxel-wise transformed using Fisher’s *r-*to-*z* transformation. The final brain maps comprise *z*-values indicating the functional connectivity strength of each voxel with respect to the defined seed-voxel^40^. The demonstrated functional connectivity networks were computed by using a threshold of |*z*| ≥ 0.4; voxels below this threshold were assumed not be functionally connected, i.e. not to be part of the respective network. Higher z-scores indicate strong BOLD synchronization between the voxel with respect to the seed-voxel which is associated with functional coupling.

The two-sided parametric unpaired Student’s *t*-test for unequal variances was used to test for voxel-wise differences between either two groups, the two-sided parametric paired Student’s *t*-test was used to test for group differences over time; *p* < 0.05 indicated statistical significance. The resulting *p*-values were FDR corrected for multiple comparisons at 5% level with further clusterwise correction discarding isolated clusters <343 mm^3^.

### DTI-based fiber tracking and statistical analysis

DTI data sets were used for tract-wise fractional anisotropy (FA) statistics in six fiber tracts that become sequentially involved in the ALS-associated pathological progress^3^. In particular, we traced FA using a seed-to-target approach^38^ with seed-target coordinates as previously defined^3^ along (1) the corticospinal tract (CST, ALS-stage 1^3^) corresponding to the motor network, (2) the corticopontine and –rubral tract (ALS-stage 2^3^) corresponding to the brainstem network, (3) the corticostriatal pathway (ALS-stage 3^3^) corresponding to the ventral attention network, (4) the proximal portion of the perforant pathway (ALS-stage 4^3^) corresponding to the default mode/hippocampal network, and (5) a reference pathway in area 5 of the corpus callosum as previously defined^3^, according to our hypothesis.

The CST as the corresponding tract for the initial neuropathological ALS-stage 1^3^ was additionally subjected to Spearman rank order correlation analyses in order to detect possible relationships with functional networks alterations. Non parametric statistics were used for statistical pairwise group comparison, i.e. Mann-Whitney-*U*-test for cross-sectional data and Wilcoxon signed rank test for testing differences over time. The CST as the corresponding tract for ALS-stage 1^3^ was used for tract-wise fractional anisotropy statistics. DTI data sets were used to compute FA for the CST using a seed-to-target approach^38^. All values were computed using the MATLAB^®^ (The Mathworks Inc, Natick, MA, USA) based ‘Statistics Toolbox’.

### Relations between network-based functional connectivity, FA, and clinical scores

Possible relationships between functional connectivity and both FA and clinical scores (i.e., ALS-FRS-R score) were studied for all ALS patients (*N* = 135) using non-parametric Spearman’s rank-order correlation coefficient. The resulting *p*-values were FDR-corrected for multiple comparisons.

## Additional Information

**How to cite this article**: Schulthess, I. *et al*. Functional connectivity changes resemble patterns of pTDP-43 pathology in amyotrophic lateral sclerosis. *Sci. Rep.* **6**, 38391; doi: 10.1038/srep38391 (2016).

**Publisher's note:** Springer Nature remains neutral with regard to jurisdictional claims in published maps and institutional affiliations.

## Figures and Tables

**Table 1:** Diffusion tensor imaging-based fiber tracing.

	Healthy controls	ALS, all, *T*_0_	*p*-value^a^	ALS, *T*_0_	ALS, *T*_1_	*p*-value^b^
Corticospinal tract	**0.372** (0.355–0.395), 0.301–0.483	**0.350** (0.327–0.366), 0.247–0.451	**<10**^−**6**^	**0.350 (**0.334–0.374), 0.290–0.451	**0.346 (**0.329–0.367), 0.279–0.437	**0.0009**
Corticopontine tract/corticorubral tract^c^	**0.374** (0.365–0.387), 0.326–0.399	**0.356** (0.342–0.371), 0.247–0.393	**<10**^**−5**^	**0.358** (0.349–0.372), 0.268–0.404	**0.355** (0.341–0.368), 0.266–0.406	**0.036**
Corticostriatal pathway	**0.307** (0.288–0.325), 0.225–0.369	**0.286** (0.256–0.305), 0.199–0.376	**<10**^**−4**^	**0.281** (0.249–0.305), 0.210–0.378	**0.273 (**0.252–0.313), 0.211–0.373	0.195
Perforant pathway	**0.192** (0.178–0.213), 0.153–0.243	**0.180** (0.168–0.196), 0.150–0.234	**<10**^**−2**^	**0.188** (0.172–0.201), 0.150–0.222	**0.186** (0.171–0.205), 0.148–0.221	0.809
Corpus callosum (area 5)	**0.354** (0.333–0.376), 0.256–0.399	**0.366** (0.328–0.378), 0.224–0.397	0.517	**0.352** (0.322–0.377), 0.286–0.400	**0.349** (0.315–0.374), 0.280–0.401	0.112

Values indicate tract-wise Fractional anisotropy (FA) and are shown as median (interquartile range), min—max.

^a^Wilcoxon Mann-Whitney-*U* test refers to comparison between all ALS patients and healthy controls.

^b^Two-sample Wilcoxon signed rank test refers to comparison between ALS patients at *T*_0_ (baseline) and *T*_1_ (follow-up) measurement.

^c^Arithmetically averaged FA values for the corticopontine tract and corticorubral tract according to^17^.

**Table 2:** Subject demographics and clinical characterization.

	Healthy controls	ALS, all, *T*_0_	*p*-value	ALS, *T*_0_	ALS, *T*_1_	Δ |*T*_1_ − *T*_0_|	*p*-value^b^
**Subjects** (number)	**56**	**135**	NA	**27**	**27**		
**Gender** (male:female)	**25** : **31**	**75** : **60**	0.203^a^	19 : 8	19 : 8		1.000
**Age** (years)	**61** (50–66), 22–76	**61** (55–70), 20–80	0.054^c^	**63.6** (57.6–68.2), 47.1–78.6	**64.0** (56.4–69.0), 48.0–79.1	**0.6** (0.4–0.8), 0.4–1.3	**<0.0001**
**Duration of disease** (month)	NA	**13** (8–24), 2–77	NA	**18** (12–38), 6–77	**26** (18–45), 16–86	**6** (5–9), 4–12	**<0.0001**
**Age of onset** (years)	NA	**60** (51–68), 19–80	NA	**63** (57–68), 46–77			
**ALSFRS-R**^d^	NA	**42** (37–45), 22–48	NA	**42** (40–44), 26–47	**39** (33–42), 20–46	**4** (2–6), 0–17	**<0.0001**
**Rate of disease progression**^e^ (1/month)	NA	**0.4** (0.2–0.9), 0.0–4.3	NA	**0.4** (0.2–0.9), 0.0–4.1	**0.4** (0.2–0.6), 0.0–1.4	**0.1** (0.0–0.3), 0.0–2.9	0.161

Data are shown as median (interquartile range), min—max.

^a^Fisher’s exact test refers to comparison between all ALS patients and healthy controls.

^b^Two-sample Wilcoxon signed rank test refers to comparison between ALS patients at *T*_0_ (baseline) and *T*_1_ (follow-up) measurement.

^c^Wilcoxon Mann-Whitney-*U* test refers to comparison between all ALS patients and healthy controls.

^d^ALSFRS-R, revised ALS Functional Rating Scale (maximum score 48, falling with increasing physical impairment).

^e^Rate of disease progression computed as (48–ALSFRS-R)/(disease duration) according to^35^. NA, not applicable.
